# Pathogenic and Potential Therapeutic Roles of Exosomes Derived From Immune Cells in Liver Diseases

**DOI:** 10.3389/fimmu.2022.810300

**Published:** 2022-02-04

**Authors:** Leyu Zhou, Mengyi Shen, Xiaoli Fan, Yifeng Liu, Li Yang

**Affiliations:** Sichuan University-University of Oxford Huaxi Joint Centre for Gastrointestinal Cancer, Frontiers Science Center for Disease-Related Molecular Network, Department of Gastroenterology and Hepatology, West China Hospital, Sichuan University, Chengdu, China

**Keywords:** exosomes, immune cells, liver diseases, biomarker, therapy

## Abstract

Liver diseases, such as viral hepatitis, alcoholic hepatitis and cirrhosis, nonalcoholic steatohepatitis, and hepatocellular carcinoma place a heavy burden on many patients worldwide. However, the treatment of many liver diseases is currently insufficient, and the treatment may be associated with strong side effects. Therapies for liver diseases targeting the molecular and cellular levels that minimize adverse reactions and maximize therapeutic effects are in high demand. Immune cells are intimately involved in the occurrence, development, and prognosis of liver diseases. The immune response in the liver can be suppressed, leading to tolerance in homeostasis. When infection or tissue damage occurs, immunity in the liver is activated rapidly. As small membrane vesicles derived from diverse cells, exosomes carry multiple cargoes to exert their regulatory effects on recipient cells under physiological or pathological conditions. Exosomes from different immune cells exert different effects on liver diseases. This review describes the biology of exosomes and focuses on the effects of exosomes from different immune cells on pathogenesis, diagnosis, and prognosis and their therapeutic potential in liver diseases.

## Introduction

Liver diseases impose a heavy burden worldwide. Approximately 2 million people die from liver diseases annually worldwide, mostly due to viral hepatitis (VH), complications of liver cirrhosis and hepatocellular carcinoma (HCC). Cirrhosis ranks 11th among the most common causes of death worldwide, while liver cancer ranks 16th. The global prevalence of VH remains high, and drug-induced liver injury (DILI) increases persistently. In addition, alcohol and obesity account for a portion of patients with liver diseases (1). Current therapies for liver diseases are insufficient to a certain degree. Some patients have a poor response to the available therapies or experience strong side effects. Therefore, studies exploring the mechanisms of pathogenesis and new therapies for liver diseases are urgently needed.

Immune cells exert an important effect on the process of disease development in the liver. Many mechanisms may be involved, such as Toll-like receptor (TLR) signaling, molecular danger patterns, and inflammasome activation. For example, CX3CL1 (one chemokine) is secreted in some chronic liver diseases, such as alcoholic liver disease (ALD), autoimmune hepatitis (AIH) and primary biliary cirrhosis (PBC). CX3CL1 mediates intermediate CD14^high^ CD16^+^ monocyte accumulation, leading to monocyte infiltration in the liver. Additionally, CD14^high^ CD16^+^ macrophages secrete profibrogenic cytokines and activate collagen-producing stellate cells, which are associated with hepatic fibrogenesis (2).

Exosomes are nanometer-sized, lipid membranes-encapsulated particles derived from almost all types of cells that are present in many bodily fluids, which protects their cargoes from being degraded by enzymes in bodily fluids (3). Furthermore, exosomes transfer their cargoes between cells or different sites in the body, thus mediating intercellular communication under physiological and pathological conditions. Therefore, materials in exosomes remain stable and are comparatively ideal for detection as biomarkers or use in the development of new therapies. Exosomes have been widely investigated in liver diseases, including ischemia/reperfusion injury (IRI) (4), VH (5), ALD (6), nonalcoholic steatohepatitis (NASH) (7), liver fibrosis (8), and HCC (9). In liver diseases, exosomes either inhibit (4) or enhance (9) disease progression and may also play roles in other peocesses. For example, exosomes derived from immune cells have been studied in liver diseases. This review provides a simple overview of exosomes and then describes how immune cell-derived exosomes affect the pathogenesis of liver diseases (**Table 1**). Next, the review summarizes the effects of exosomes on pathogenesis, diagnosis and prognosis, providing a perspective on the therapeutic potential of exosomes derived from immune cells in liver diseases.

**Table 1:** Summary of the effects of exosomes derived from immune cells on liver diseases.

	Source	Target cells	Mechanism	Effect	References
Monocytes	THP-1 monocytes (treated with alcohol)	Naïve monocytes	Exosomal miR-27a enhanced polarization by targeting CD206	Naïve monocytes polarized into M2 macrophages	(10)
Macrophages	RAW 264.7 macrophages (stimulated with LPS)	Hepatocytes	Exosomes mediated NLRPS signaling pathway activation	Hepatocyte damage, inflammatory cell infiltration and liver injury	(11)
	RAW 264.7 macrophages (treated with Con A)	RAW 264.7 macrophages	Exosomal mmu-miR-122-5p and mmu-miR-148a-3p were transferred and targeted the ROCK1 gene	Inflammatory cytokine production by Con A-stimulated RAW 264.7 cells was suppressed	(12)
	THP-1 macrophages (stimulated with LPS)	HSCs	Exosomal miR-103-3p targeted the 3’UTR of KLF4	HSC proliferation and activation were promoted	(13)
	Macrophages (treated with IL-6)	Hepatocytes	Exosomes mediated miR-223 transfer and reduced pro-fibrotic TAZ expression in hepatocytes	NAFLD-associated fibrosis was attenuated	(14)
	Macrophages (treated with relaxin)	HSCs	Exosomal miR-30a-5p suppressed ASK1 expression and revive the relaxin-mediated PPAR-γ activation and antifibrotic effect on aHSCs	Antifibrotic effect on aHSCs and liver fibrosis amelioration	(15)
	THP-1 macrophages (stimulated with IFN-α)	Hepatocytes (HepG2.2.15 and HepG2 cells)	Exosomes mediated the intercellular transfer of antiviral molecules	IFN-α-induced antiviral activity was transmitted to hepatocytes	(5, 16)
	TLR3-activated macrophages (treated with poly I:C)	Hepatocytes (Huh7 cells)	Exosomes mediated the delivery of anti-HCV miRNA-29 family members.	HCV replication in Huh7 cells was inhibited	(17)
	TREM2-deficient macrophages (treated with PA)	Hepatocytes	Exosomal miR-106b-5p inhibited Mfn2 and caused mitochondrial dysfunction	The NAFLD progression was enhanced and NAFLD -associated sepsis aggravated *in vivo*,	(18)
	THP-1 macrophages (induced by PMA)	Liver cancer SK-HEP-1 and HA22T cells	Exosomal miR-92a-2-5p altered the AR/PHLPP/p-AKT/β-catenin signaling pathway	The invasion capacity of liver cancer cells increased	(19)
	TAMs	HCC cells (Huh7, HepG2 and BEL-7404 cells)	MiR-125a/b targeted the 3’UTR of CD90 mRNA to downregulate CD90 and the level of miR-125a/b in TAMs is low	HCC cell proliferation and stem cell properties were enhanced	(20)
	THP-1 macrophages (treated with arsenite)	HCC cells	Exosomal miR-15b regulated the Hippo pathway by targeting LATS1	The progression of hepatocellular carcinoma was increased	(21)
	M2-TAMs	HCC cells	The imbalance of TGF-β1/BMP-7 pathways was induced	HCC progression was enhanced	(22)
DC	BMDCs	Naïve T cells	DEXs mediated the balance of Tregs and Th17 cells by transporting HSP70 and stimulating the PI3K/mTOR axis	Hepatic ischemia-reperfusion injury was alleviated	(23)
	Bone marrow imDCs	Tregs	imDEXs amplified Tregs through a mechanism requiring recipient DCs, and Tregs retained donor specificity	Tolerance was induced and long-term survival was achieved in a rat liver -allograft model	(24)
	AFP-expressing DCs (DC2.4 cells)	T cells	DEX_AFP_ induced the AFP-specific activation of T cells and triggered potent antigen-specific antitumor immune responses	Improvement in the tumor microenvironment and suppression of tumor growth	(25)
NK	NK-92MI cells	HSCs (LX-2 cells)	Exosomal-mediated transfer of miR-223 inhibited autophagy by targeting ATG7	TGF-β1-induced HSC activation and CCl_4_-induced liver fibrosis were inhibited	(26, 27)
Neutrophils	Neutrophils	Hepatocytes	miR-223 was transferred by EVs to hepatocytes with selective control by LDLR and APOE	Hepatic inflammatory and fibrogenic gene expression was inhibited and nonalcoholic steatohepatitis was ameliorated	(28)
T cells	CD4^+^CD25^+^ Tregs	CD8^+^ CTLs	Exosomes mediated the inhibitory effect on CD8^+^ CTL proliferation and viability by downregulating the mRNA levels of IFN-γ and perforin	Liver allograft survival was prolonged after liver transplantation in an OLT rat model	(29)
B cells	B cells	Hepatocytes	Exosomes transferred miR-155 to hepatocytes and rituximab decreased B cell–derived exo‐miR‐155 levels.	HCV replication was inhibited in hepatocytes while rituximab restrained this process in patients with RA	(30)
Mast cells	HMC-1 cells (stimulated with HCV-E2)	Hepatocytes (HepG2 cells)	Exosomes inhibited the ERK1/2 pathway by transferring miR-490 into HCC cells	HCC cell migration and metastasis were inhibited	(31)

LPS, lipopolysaccharide; Con A, concanavalin A; HSCs, hepatic stellate cells; KLF4, Krüppel-like factor 4; TAZ, transcriptional activator with PDZ-binding motif; NAFLD, nonalcoholic fatty liver disease; ASK1, apoptosis signal-regulating kinase 1; PPAR-γ, peroxisome proliferator-activated receptor-γ; aHSCs, activated hepatic stellate cells; TLR3, Toll-like receptor 3; Poly I:C, polyinosinic-polycytidylic acid; HCV, hepatitis C virus; TREM2, triggering receptor expressed on myeloid cells-2; PA, palmitic acid; Mfn2, mitofusin 2; PMA, phorbol-12-myristate-13-acetate; TAMs, tumor associated macrophages; HCC cells, hepatocellular carcinoma cells; LATS1, large tumor suppressor kinase 1; M2-TAMs, M2-polarized tumor-associated macrophages; BMDCs, bone marrow-derived dendritic cells; DEXs, exosomes produced by BMDCs; HSP70, heat shock protein 70; imDCs, immature dendritic cells; imDEXs, exosomes from imDCs; AFP, α-fetoprotein; DEXAFP, exosomes derived from α-fetoprotein (AFP)-expressing DCs; NK cells, Natural killer cells; ATG7, autophagy-related protein 7; LDLR, low-density lipoprotein receptor; APOE, apolipoprotein E; CTLs, cytotoxic T lymphocytes; OLT, orthotopic liver transplantation; RA, rheumatoid arthritis.

## Biogenesis and Functions of Exosomes

**Figure 1:**
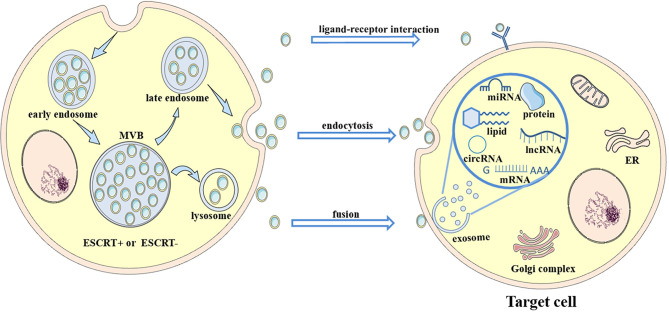
Biogenesis and secretion of exosomes. Multivesicular bodies (MVBs) are formed by plasma membrane invagination followed by process in the rough endoplasmic reticulum (RER) and Golgi body. Then, some MVBs fuse with the plasma membrane to release small vesicles called exosomes to extracellular environment, while some MVBs fuse with lysosomes and are degraded. Exosomes carry abundant cargoes to target sites, such as proteins, lipids and RNAs. Exosomes affect target cells through ligand-receptor interactions, endocytosis or fusion with the plasma membrane of target cells. After an interaction with target cells, exosomes (along with their contents) alter some signaling pathways and target genes, change their expression levels in target cells, and transfer antiviral molecules or other substances into target cells. Thus, the disease condition is aggravated or alleviated. For example, exosomes transfer the CD11/CD18 protein from TAMs to HCC cells and activate the MMP-9 signaling pathway in HCC cells. Then, the migratory potential of HCC cells is increased.

Extracellular vesicles (EVs) are divided into two major subtypes that are defined based on their biological pathways: microvesicles and exosomes (32–38). Microvesicles are generated by plasma membrane budding, while exosomes are generated after the fusion of multivesicular endosomes (MVEs) and the plasma membrane (32). Exosomes are nanosized vesicles with diameters ranging from ~30 to ~200 nm (39). They are secreted outside the cell and carry abundant proteins, lipids, and nucleic acids that vary in different cases. EVs target their recipient cells *via* their surface molecules. They trigger signaling through receptor-ligand interactions or after fusion with the plasma membrane or endocytosis/phagocytosis to release their cargoes into target cells, therefore modifying the physiological state of these target cells (40). Multiple cells release exosomes, and exosomes in the blood are mixed populations; thus, the different biogenesis pathways of exosomes in different states remain to be explored (**Figure 1**).

Exosomes mediate cell-to-cell communication by delivering their cargoes to recipient cells or fusing with the plasma membrane, which potentially alters recipient cells signaling, gene expression, and overall function (41). For instance, immature dendritic cells (imDCs) transfer functional major histocompatibility complex MHC-peptide complexes to other DCs by secreting exosomes to activate the immune response (42). MicroRNAs are transferred unidirectionally from activated T cells to antigen-presenting cells *via* exosomes (43). As exosomes encapsulate therapeutic mRNAs, targeting peptides or other molecules, they are also widely used as vesicles for drug delivery (44). Hepatitis A virus (HAV) infection is mediated by exosome mimicry and a subsequent increase in content delivery from the exosomes of HAV-infected cells (45). Formed from the lipid bilayer membrane, exosomes protect their cargo from degradation by enzymes in the circulation, and thus that the detection of exosomal cargoes presents a potential method for disease diagnosis and estimation of the therapeutic effect of a specific drug (3, 46, 47), such as exosomal α-synuclein for Parkinson’s disease (48). Different mechanisms and pathways related to exosome uptake and the specificity of exosomes from certain cell types increase the complexity of their functions in intercellular communication (37, 49, 50).

## Exosomes Derived From Immune Cells in Liver Diseases

### Monocyte- and Macrophage-Derived Exosomes

Monocytes are thought to originate from hematopoietic stem cell-derived progenitors. They produce subsets of inflammatory DCs and macrophages after exiting blood vessels and entering tissues under inflammatory conditions. Their functions comprise antigen- presentation, tissue remodeling, and inflammatory cytokine production, among others (51, 52). Derived from monocytes, macrophages are generated after monocytes leave the vessels and enter connective tissue; these cells are characterized by increased numbers of lysosomes and enhanced phagocytic function when they are exposed to pro-inflammatory cytokines, local growth factors, and microbial products. Macrophages perform various functions: 1) phagocytosis of pathogens, dead cells and debris; 2) antigen presentation by exposing the processed antigen to MHC molecules; and 3) production of distinct types of cytokines, such as TNF‐α, IL-1, and IL‐6 (53, 54).

#### Acute Liver Injury

Acute liver injury (ALI) is induced by many factors, and a previous study investigated sepsis-induced liver injury. The study showed that some inflammation-associated proteins were upregulated in exosomes derived from LPS-induced macrophages, such as CCL3, CCL22, CXCL2, CXCL10, CD40, and TNF. Serum ALT, AST, and LDH levels were increased, and extensive inflammatory cell infiltration was observed in the livers of mice treated with these exosomes. Exosomes from LPS-induced macrophages were mainly taken up by hepatocytes and the NLRP3 signaling pathway was activated in hepatocytes, which are related to the process of sepsis-induced ALI. This communication between macrophages and hepatocytes mediated by exosomes may prompt us to identify macrophage exosomes as therapeutic targets for sepsis (11). Another study revealed that EVs derived from concanavalin A (Con A)-treated macrophages were taken up by macrophages and suppressed the production of inflammatory cytokines in EV-pretreated- Con A-stimulated macrophages. These EVs contained large amounts of mmu-miR-122-5p and mmu-miR-148a-3p. The mRNA levels of ROCK 1, a target gene of these two miRNAs, were significantly decreased by EV pretreatment. The ROCK 1 gene is related to the Rho/ROCK pathway, which regulates the production of inflammatory cytokines. These data showed the immunoregulatory role of macrophage-derived EVs in Con A-induced hepatitis (12).

#### Viral Hepatitis

Macrophage-derived exosomes transfer interferon-α (IFN-α)-induced anti-HBV activity to hepatocytes, and a study showed how exosomes enter hepatocytes to complete this delivery. They found that PtdSer (an apoptosis marker) was expressed on the outer membrane of macrophage-derived exosomes. A receptor of PtdSer, T cell immunoglobulin and mucin receptor 1 (TIM-1), uniquely mediated exosome internalization by hepatocytes. In this process, clathrin-mediated endocytosis and macropinocytosis collaborate to permit exosome entry. Then, membrane fusion of exosomes in late endosomes/multivesicular bodies occurred to release exosomal cargo for efficient delivery of IFN-α-induced anti-HBV activity (16). Antiviral molecules are also transferred from IFN-α-treated macrophages or liver sinusoidal cells to hepatocytes through the internalization of exosomes by hepatocytes and then reduce HBV replication in a hepatitis B model (5). Similarly, TLR3-activated macrophages delivered exosomes containing increased levels of anti-HCV miRNA‐29 family members. They were taken up by hepatocytes, and the replication of hepatitis C virus (HCV) was inhibited. Therefore, antiviral miRNAs were transferred from macrophages to hepatocytes, where an anti-HCV response was induced or HCV gene expression was targeted directly (17), which suggests a potential therapy for HCV. Another study determined that EVs from interferon-stimulated macrophages inhibit HCV replication and that this EV-mediated antiviral immune response was hindered by the presence of polyunsaturated fatty acids (55).

#### ALD and Nonalcoholic Fatty Liver Disease

In one study, an analysis of circulating EVs from the plasma of patients with alcoholic hepatitis (AH) showed increased numbers of EVs that contained large amounts of miR-27a compared with healthy people, which may imply the potential of exosomal miR-27a to serve as a diagnostic biomarker of AH. Additionally, alcohol-treated monocytes released EVs (mainly exosomes) in a concentration- and time-dependent manner, which contained large amounts of miR-27a. MiR-27a in EVs stimulates naïve monocytes to polarize into M2 macrophages and increases IL-10 and TGF-β release and phagocytosis activity by targeting CD206, suggesting its role in the pathogenesis of alcohol-associated liver diseases (10). In individuals with nonalcoholic fatty liver disease (NAFLD), miR-106b-5p is upregulated in exosomes derived from TREM2-(triggering receptor expressed on myeloid cells-2)-deficient macrophages. It blocks mitofusin 2 (Mfn2), which is important for mitochondrial fusion, thus impairing mitochondrial function and affecting the energy supply of hepatocytes. Finally, it promotes NAFLD progression and aggravates NAFLD-associated sepsis *in vivo*. This result suggests the pathogenic role of miR-106b-5p in NAFLD and the potential of TREM2 in precision treatment (18). Additionally, exosomes isolated from the supernatants of macrophages exposed to LPS induce a metainflammation phenomenon (56). Another study showed that EVs derived from macrophages correlated with the histological NAS (NAFLD activity score), which indicates the severity of NASH. Circulating macrophage-derived EVs may serve as novel biomarkers for the diagnosis and follow-up of NASH (57). Additionally, adipose tissue macrophages (ATMs) in obese mice were shown to release exosomes containing overexpressed miR-155. These exosomes are taken up by insulin target cells and cause cellular and systemic insulin resistance and glucose intolerance. These changes may be attributed to a mechanism most likely related to direct suppression of PPARγ, a target gene of miR-155. Furthermore, exosomes derived from ATMs in lean mice improved glucose tolerance and insulin sensitivity after administration to obese mice (58). The results of this trial may provide insights into new therapeutic options for metabolic liver diseases.

#### Liver Fibrosis

Macrophages stimulated with LPS also participate in the pathogenesis of hepatic fibrosis. A study reported higher miR‐103‐3p expression in circulating exosomes from patients with fibrotic stages S3-S4 than in exosomes from patients with early-stage disease, indicating that miR‐103‐3p from circulating exosomes may be a biomarker for the diagnosis and grading of hepatic fibrosis. A subsequent study reported that miR‐103‐3p, which is present at increased levels in exosomes from LPS‐treated THP‐1 macrophages, promotes the proliferation and activation of hepatic stellate cells (HSCs) by targeting the 3’UTR of Krüppel-like factor 4 (KLF4), thus exacerbating the progression of liver fibrosis (13). According to another study, IL-6 promotes the release of miR-223-enriched exosomes from palmitic acid (PA)-treated macrophages, which are transferred into hepatocytes and target transcriptional activator with PDZ-binding motif (TAZ) in hepatocytes. Thus, NASH fibrosis is inhibited. Therefore, myeloid-specific IL-6 signaling inhibits liver fibrosis by transferring antifibrotic miR-223 into hepatocytes *via* exosomes (14). Furthermore, hepatic macrophages switch from a profibrogenic to a pro-resolution phenotype through Nur77 (an orphan nuclear receptor) activation after treatment with relaxin. Then, these macrophages release exosomes containing miR-30a-5p that are taken up by activated HSCs (aHSCs). Moreover, miR-30a-5p suppresses apoptosis signal-regulating kinase 1 (ASK1) and activates peroxisome proliferator-activated receptor-γ (PPAR-γ), to promote the relaxin-mediated quiescence of aHSCs and leads to an antifibrotic effect on aHSCs. Based on the results, the authors developed lipid nanovesicles containing the relaxin gene and miR-30a-5p mimic, which preferentially targets aHSCs. The antifibrotic effect is enhanced on multiple liver fibrosis models (15). This strategy may provide a new idea for the potential therapy of liver fibrosis.

#### HCC

A previous study showed an increase in miR-92a-2-5p expression in exosomes from macrophages in liver cancer, which directly targets the 3’UTR of the androgen receptor (AR) mRNA to decrease its expression in liver cancer cells. A reduction in AR expression increases the invasion of liver cancer cells by altering PHLPP/p-AKT/β-catenin signaling (19). Another study found that exosomes derived from tumor-associated macrophages (TAMs) contain low levels of miR-125a and miR‐125b, which promote HCC cell proliferation, migration and stem cell properties by targeting CD90 (20). Additionally, miR-21-5p is upregulated in EVs derived from M2 macrophages and is transported into the liver tissue in mice. M2 macrophage-derived EVs are taken up by CD8^+^ T cells and facilitate CD8^+^ T cell exhaustion by targeting YOD1, which inactivates the YAP/β-catenin pathway. Thus, tumor progression in HCC mice is exacerbated through the miR-21-5p/YOD1/YAP/β-catenin axis (59). Moreover, THP-1-derived macrophages polarize into M2 macrophages after treatment with arsenite in a concentration-dependent manner. Then, miR-15b in EVs released by these macrophages is transferred into HCC cells and targets large tumor suppressor kinase 1 (LATS1) to regulate the Hippo pathway, thus increasing the proliferation, migration, and invasion of HCC cells (21). Similarly, EVs derived from M2 polarized tumor-associated macrophages (M2-TAMs) containing MIR17HG and miR-17-92 cluster components are taken up by HCC cells, which induce an imbalance in TGF-β1/BMP-7 pathways, thus enhancing HCC progression (22).

### DC-Derived Exosomes

DCs are derived from bone marrow progenitors through lympho-myeloid hematopoiesis. DCs are divided into two types: mature DCs and immature DCs. Mature DCs induce and regulate the immune response to foreign antigens through their antigen capture and processing abilities, whereas imDCs induce self-tolerance. DCs, as well as their exosomes, are involved in many processes associated with transplantation, atherosclerosis, infectious diseases, autoimmune disorders and cancer (60–63). Exosomes derived from DCs carry MHC-peptide complexes, facilitating the effective activation of T cells. Other studies have shown EVs released from imDCs exert different effects on T cells (51, 64–69).

#### Non Neoplastic Liver Diseases

A previous study showed that a large quantity of exosomes is secreted by bone marrow-derived dendritic cells (BMDCs) exposed to supernatants from hypoxic and reoxygenated(H/R) primary hepatocytes. These exosomes carry HSP70 and are taken up by naïve T cells, which modulate the differentiation of Tregs and Th17 cells through the PI3K/mTOR axis. Modulation of the balance between Tregs and Th17 cells by BMDCs alleviates liver IRI. The injection of exosomes derived from BMDCs (DEXs) improves liver function in mice after IR. The data described above suggest the potential clinical application value of DEXs (23). In addition, exosomes from immature DCs (imDEXs) amplify Tregs both *in vivo* and *in vitro*. The amplified Tregs retain donor specificity and their regulatory ability. The administration of donor imDEXs combined with donor-specific Tregs decreases the levels of infiltrating cells and rejection symptoms in rat liver allografts and assists with the regeneration of recipient livers after experiencing slight acute rejection, thus prolonging liver allograft survival. This tolerance may be induced by imDEXs and donor-specific Tregs without the need for immunosuppressive agents, which might avoid the side effects of these suppressants. This finding may indicate the potential of imDEXs in clinical therapy (24).

#### HCC

DC-derived exosomes also play an important role in HCC. A previous experiment showed that microwave ablation combined with Dex enhances antitumor efficacy and significantly inhibits tumor growth, as well as improves the immune microenvironment compared with microwave ablation alone (70). Alpha fetoprotein (AFP) is an oncodevelopmental protein (71) and a clinical biomarker of HCC. AFP-expressing DC-derived exosomes (DEX_AFP_) promote strong antigen-specific immune responses that inhibit tumor growth and reshap the tumor microenvironment. The antitumor effect mediated by DEX_AFP_ positively correlates with the ameliorated immune microenvironment in HCC mice, and T cells contribute to the antitumor function of DEX_AFP._ Based on these results, DEX_AFP_ has potential clinical value for cancer immunotherapy as a novel class of vaccines. However, its application potential remains unclear. The process of DC maturation is dynamic *in vivo*; thus, extracted exosomes may be derived from a mixture of DCs with different levels of maturity. The dose and administration route of DEX_AFP_ must be optimized in clinical practice. MHC I- and MHC II-restricted antigens of DEXs in patients may be inadequate to elicit tumor-targeted T cell responses, and the application of mutated neoepitopes of DEXs may be warranted. As research and development advance, DEXs might be used as a therapy in the near future (25, 72).

### Natural Killer Cell and Neutrophil-Derived Exosomes

Natural killer (NK) cells are large granular lymphocytes that are components of the innate immune system and exert cytotoxic and cytokine-producing functions (73, 74). NK cells residing in different tissues display distinct phenotypic profiles and perform different functions (75). Neutrophils are presumed to be constituted of a population of homogeneous cells containing different subpopulations under physiological and pathological conditions. Neutrophils perform antimicrobial functions through degranulation, phagocytosis, reactive oxygen species release, and the production of antimicrobial proteins and neutrophil extracellular traps (NETs) (76, 77). NK cells and neutrophils, as well as their exosomes, are involved in many diseases, such as infection, inflammation and cancer (78–82).

NK cells have been confirmed to be involved in liver fibrosis (83–88). In a previous study, NK-Exos were taken up by HSCs after an incubation with NK-Exos and TGF-β1-treated HSCs. A remarkable reduction in the cell proliferation rate and expression of CoL1A1 and α-SMA was subsequently observed in HSCs, inhibiting the activation and proliferation of TGF-β1-treated HSCs. In mice, the administration of NK-Exos decreased the serum AST and ALT levels and alleviated CCl_4_-induced liver injury and fibrosis by decreasing the mRNA levels of profibrogenic genes. This result suggests that exosomes from NK cells may represent novel treatments for liver injury or fibrosis in the future (26). A subsequent study explored the mechanism underlying the effects of NK-Exos on liver fibrosis. Abundant miR-223 was present in NK-Exos. And NK-Exos attenuated TGF-β1-induced HSC activation by transferring miR-223 to suppress TGF-β1-induced autophagy by targeting and inhibiting ATG7 expression in HSCs (27).

Neutrophil infiltration around lipotoxic hepatocytes is one characteristic of NASH. A previous study showed that neutrophil-derived EVs are related to histological changes in NASH and the fibrosis stage (75). According to another study, hepatocytes assimilate EVs with abundant miR-223 from neutrophils through a mechanism that partially depends on the expression of apolipoprotein E (APOE) on EVs from neutrophils and low-density lipoprotein receptor (LDLR) on hepatocytes. The expression of hepatic inflammatory and fibrogenic genes is subsequently inhibited, thus ameliorating NASH. However, the lack of EV-derived miR-223 assimilation might accelerate NASH progression (from steatosis to NASH). The results described above suggest that the transport of miR-223-enriched EVs from neutrophils to hepatocytes with selective control by LDLR and APOE may be a potential therapy for NASH (28). Another experiment showed that miR-223-enriched EVs from neutrophils is absorbed by activated HSCs, thus increasing miR-223 levels in HSCs. Moreover, miR-223 inhibits HSC activation and proliferation by directly suppressing Gli2 and Pdgfra/b expression. In hepatocytes, miR-223 inhibits TAZ expression to decrease IHH secretion, inhibiting the Hedgehog signaling pathway in HSCs. Therefore, steatosis to NASH progression is hindered, and liver fibrosis is inhibited. This information may provide a new idea for the treatment of liver fibrosis (89).

### T Cell- and B Cell-Derived Exosomes

T cells and B cells originate from progenitors in the bone marrow; then, T cells migrate to the thymus for maturation and selection and then move to the periphery, and B cells begin to develop in the bone marrow and functionally mature in secondary lymphoid tissue. T cells and B cells are the core of adaptive immunity, in which T cells mainly mediate cellular immunity and B cells mainly mediate humoral immunity by producing antibodies (90, 91). Exosomes from T cells affect the pathogenesis and therapeutic development of diseases (92, 93). The function of CD8^+^ T cells will gradually deteriorate when they are persistently exposed to antigens and/or inflammatory signals under the condition of chronic infection or cancer; this state is called exhaustion (94).

Exosomes released by exhausted CD8^+^ T cells are absorbed by nonexhausted CD8^+^ T cells and subsequently damage recipient cell function, thus impairing the anticancer function of normal CD8^+^ T cells (95). And exosomes derived from CD4^+^CD25^+^ Tregs inhibit CD8^+^ cytotoxic T cell proliferation and reduce their viability by downregulating the expression of the IFN-γ and perforin mRNAs in a concentration-dependent manner. The administration of exosomes from CD4^+^CD25^+^ Tregs prolongs survival after liver transplantation in an orthotopic liver transplantation (OLT) rat model (29).

An *in vitro* experiment showed that rituximab downregulates the level of miR-155 in exosomes derived from B cells. MiR-155 from B cells inhibits HCV replication in hepatocytes through exosome transmission. HCV replication activity is remarkably increased in replicon cells treated with exosomes derived from rituximab-treated B cells. Additionally, the serum level of miR-155 in exosomes decreased in HCV-infected patients with rheumatoid arthritis (RA) who were treated with rituximab, which may cause hepatitis C viremia (30). Another study used B cell-derived exosomes as vesicles to deliver miR-155 mimics or inhibitors, and treatment of RAW macrophages with miRNA-155 inhibitor-loaded exosomes led to a significant reduction in LPS-induced TNF-α production. This study reminds us of approaches designed to modify target molecules, suggesting the potential of B cell-derived exosomes as vectors for treatment (96).

### Mast Cell-Derived Exosomes

**Figure 2:**
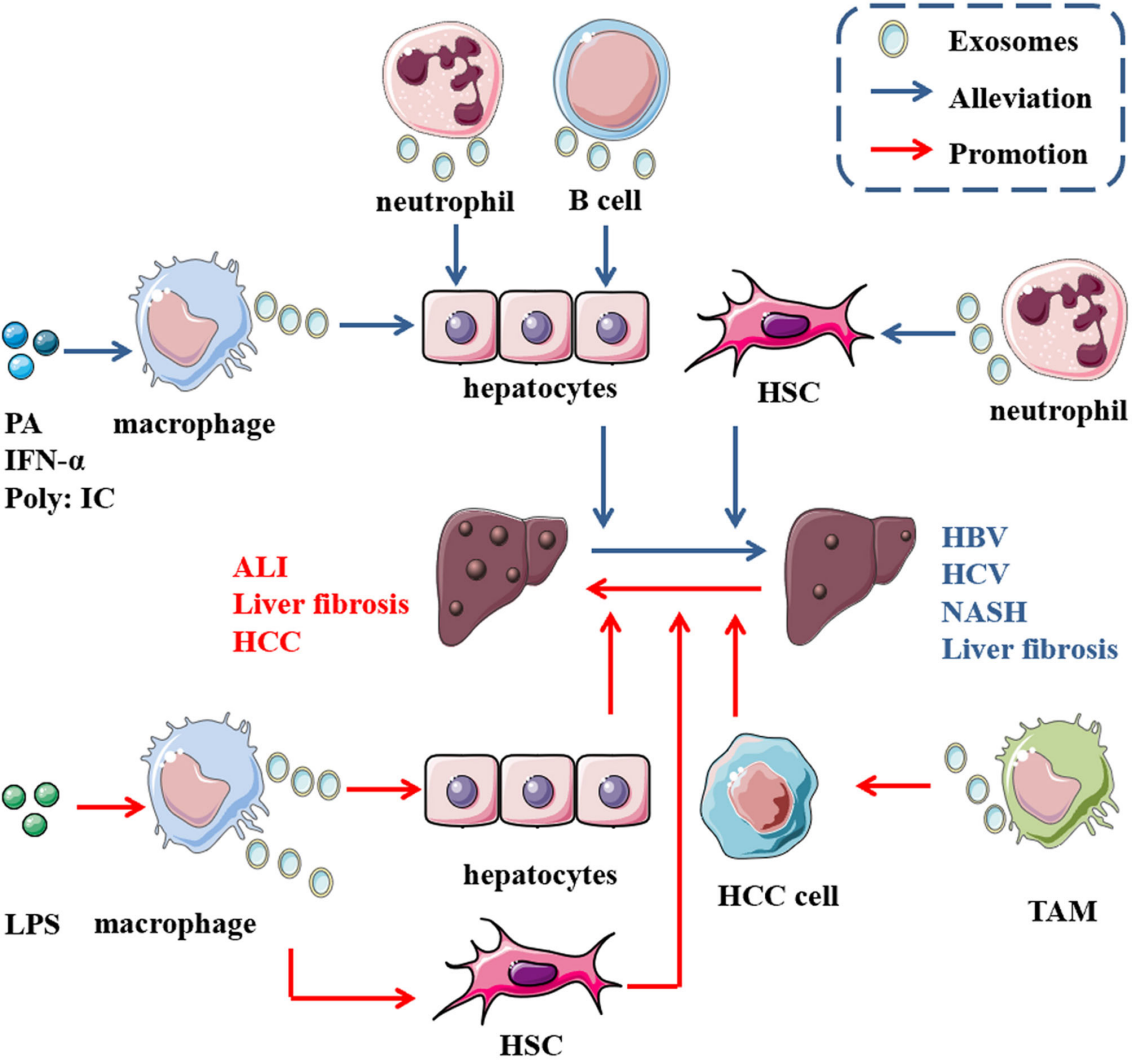
Role of exosomes derived from immune cells in liver diseases. In the presence of some molecules, immune cells release exosomes carrying multiple cargoes to act on some cells in the liver, such as hepatocytes, HSCs and liver cancer cells, thus promoting the progression of (the red arrow) or alleviating (the blue arrow) some diseases in the liver. For instance, abundant miR-223 encapsulated in neutrophil-derived EVs can be taken up by both hepatocytes and active HSCs, thus ameliorating NASH and hindering steatosis to NASH progression. Additionally, miR-155 from B cells inhibits HCV replication in hepatocytes through exosome transmission.

Mast cells are derived from hematopoietic progenitors and contain granules that participate in allergic reactions mediated by IgE (97). Exosomes derived from monocytes and mast cells are involved in disease pathogenesis (98, 99).Treatment of mast cells (MCs) with HCV-E2 envelope glycoprotein (HCV-E2) promotes the secretion of exosomes. Exosomes transfer miR-490 to recipient HepG2 cells, and then the ERK1/2 pathway in HepG2 cells is inhibited. Transfection with antagomiR-490 downregulates the level of miR-490 in exosomes derived from MCs and recipient HepG2 cells and enhances the migration of HepG2 cells, indicating that miR-490 in MC-derived exosomes participates in regulating HepG2 cell migration (31) (**Figure 2**).

## Roles of Exosomes in Diagnosis, Prognosis and Therapy

### Diagnosis

Many liver diseases are diagnosed based on their histological characteristics through a liver biopsy, which may increase the burden on patients. Thus, noninvasive markers for diagnosis are in high demand. Exosomes have been identified as potential diagnostic markers for many liver diseases. A study showed significantly increased exosomal miRNA-122a-5p levels in the circulation in a period following either acetaminophen or thioacetamide injury, which suggests its potential for use in the diagnosis of acute liver injury (ALI) induced by these drugs (100). Another study also indicated that exosomal miRNAs serve as potential biomarkers to detect drug-induced liver injury (DILI) (101). Significantly elevated miR-155 levels were detected in circulating exosomes from the sera of patients with AH compared with those of control subjects, implying its potential for use in the diagnosis of AH (102). Serum exosomal miR-103-3p levels are increased in patients with liver fibrosis. And serum exosomal miR-103-3p levels are also increased in patients with S1-S4 disease compared with patients with S0 disease. Additionally, patients with S3-S4 disease present higher miR-103-3p expression in serum exosomes than those from the hepatic fibrosis (HF) group in the early stage. The results suggest the role of serum exosomal miR-103-3p in the diagnosis and grading of liver fibrosis (13). Exosomes isolated from the plasma of patients with ALD-cirrhosis with or without HCC contained higher levels of miR-19b and miR-92 than those from healthy controls, suggest that miR-19b and miR-92 from plasma exosomes represent noninvasive biomarkers to detect acholic cirrhosis (103). Another study also suggested that miR-21-5p carried in EVs is a biomarker to detect HCC (104). Exosomes released from immune cells may also play a role in the diagnosis of liver diseases. As stated above, monocytes secreted more EVs after exposure to alcohol, and miRNA profiling revealed high miR-27a expression in these EVs. An analysis of circulating EVs from the plasma of patients with AH revealed more EVs with high levels of miR-27a than in healthy people. This result may show the potential role of exosomal miR-27a in the diagnosis of AH (10). Additionally, patients with chronic hepatitis C have increased levels of EVs derived from T cell and the levels correlate with disease severity. Patients with NAFLD have higher concentrations of EVs derived from T cells and monocytes than healthy controls, which suggests their potential diagnostic utility (105). And a study documented the presence of a possible vesicle-bound, macrophage-derived biomarker, EV-CD206, in plasma. A higher level of EV-CD206 was detected in the plasma of patients with alcoholic cirrhosis than in healthy controls, which may facilitate the diagnosis of cirrhosis (106).

### Prognosis

Exosomes may also aid in determining the prognosis of patients with liver diseases. Lipotoxic hepatocytes release miR-192-5p-enriched exosomes, and the serum miR-192-5p level positively correlates with the progression of NAFLD. This finding suggests that serum exosomal miR-192-5p potentially represent a noninvasive biomarker for monitoring NASH (107). A study showed that a higher EV concentration (higher than 5.38 x 10^11^ EVs/ml) predicted a higher mortality risk in patients with AH (108). As stated above, serum exosomal miR-103-3p from macrophages also represent a potential biomarker for the progression of liver fibrosis (13). Another study also found that exosomal miR-155 is closely associated with the progression of cirrhosis and its expression gradually increases with the severity of hepatic necrosis and fibrosis (109). High lncRNA-ATB and miRNA-21 levels in circulating exosomes, together with high C-reactive protein levels and a large tumor size, are independent predictors of disease progression and mortality in patients with HCC; thus, these molecules may serve as prognostic biomarkers of HCC (110). Another study revealed a correlation between decreased miR-16 levels and the overall survival of patients with liver cirrhosis, and high exosomal miR-192 expression is related to shorter overall survival in all patients with HCC (111).

Although many studies suggest that exosomes may represent noninvasive biomarkers for many diseases (112, 113), the results of individual studies are inconsistent. Different methods of EV purification may contribute to this discrepancy (114). Exosomes can be purified using different methods, all of which have merits and limitations. For example, early detection of cancer is usually important for determining the prognosis of patients with cancer. However, this process requires the biomarker to be identified and reliable. Exosomes obtained using different methods may have different purities, quantities, qualities and vesicle integrity. Even the same method may produce different results when different parameters are used, which may lead to misleading results and conclusions of the downstream analyses (115). Another study also showed that the exosome-EV isolation method exerts a significant effect on the analysis of the miRNAs they capsulate, which would affect the use of exosomes as clinical biomarkers (116). Since many investigation methods have been used to detect exosomes, the sensitivity and specificity of the tests must also be determined under specific conditions.

### Therapy

In addition to biomarkers for diagnosis and prognosis, exosomes also show potential in therapy. For instance, microRNAs were found to be enriched in exosomes from patients with HCV viremia and were associated with HCV-related immunopathogenesis. Direct-acting antiviral therapy markedly decrease their levels. Testing these microRNAs may reflect the response to treatment of patients with HCV (117). EVs have also been used for vaccine development, and the antigen presentation ability of DC-derived EVs to the immune system has been exploited in EV-based vaccinations (118). Although some clinical studies indicate the potential value of exosomes in clinical applications (119–121), more multicenter clinical trials are needed to validate their safety and efficacy and to lay the foundation for future clinical treatments. The possible functions of exosomes in liver disease therapy are described below.

#### Nonneoplastic Liver Diseases

Exosomes derived from immune cells may represent a possible treatment for many liver diseases. For example, treatment of obese mice with lean ATM-derived exosomes improves insulin resistance, which may inspire the development of insulin resistance therapy (58). As described above, exosomes produced by BMDCs transfer HSP70 to naïve T cells and alleviate liver IRI, as confirmed by injecting DEXs into mice with IR (23). Additionally, the transfer of miR-223-enriched EVs from neutrophils to hepatocytes can inhibits the expression of hepatic inflammatory and fibrogenic genes, thus ameliorating NASH. A lack of miR-223 may accelerate NASH progression (from steatosis to NASH, and vice versa). This finding also suggests a potential therapy for NASH (28). In addition, exosomes derived from NK cells transfer miR-223 to HSCs. Administration of these NK-Exos decreases the serum AST and ALT levels and alleviates CCl4-induced liver injury and fibrosis in mice (26, 27). Additionally, the utilization of donor imDEXs combined with donor-specific Tregs reduces the levels of infiltrating cells and rejection symptoms in liver allografts and collaboratively induce graft tolerance (24). Furthermore, some researchers developed lipid nanovesicles that encapsulate the relaxin gene and miR-30a-5p mimic based on the results of their own experiments, and these molecules preferentially target aHSCs in the fibrotic liver to finally alleviate liver fibrosis (15).

#### HCC

**Figure 3:**
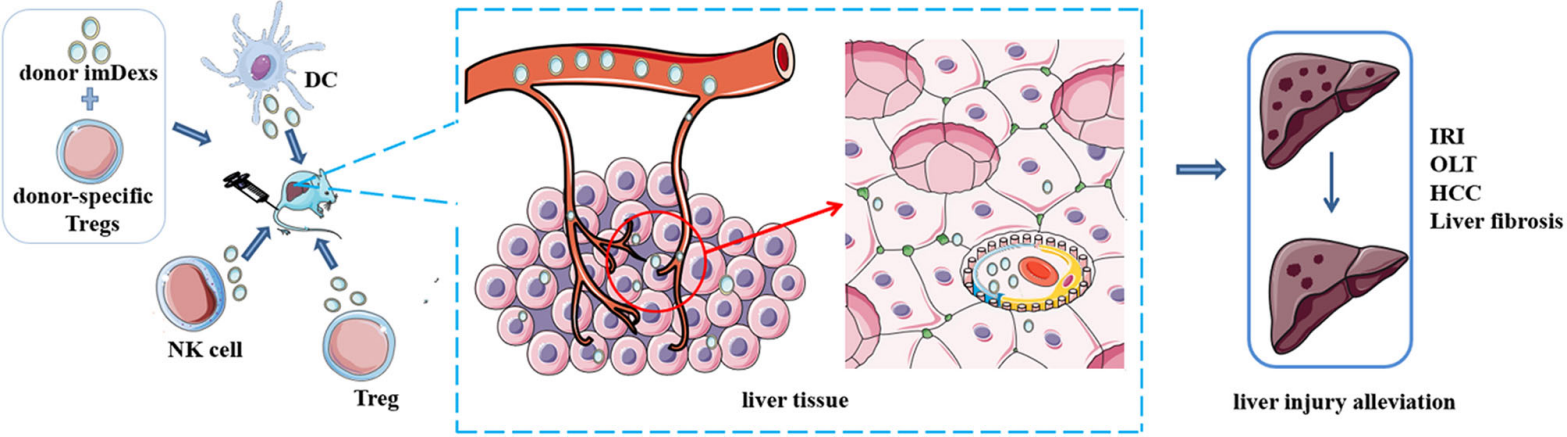
Potential of immune cell-derived exosomes in therapy. Administration of exosomes from some immune cells with or without other treatments alleviate disease condition in mice, such as ischemia/reperfusion injury, liver fibrosis and hepatocellular carcinoma. These results indicate the potential value of immune cell derived exosomes in clinical therapy.

As a new class of vaccines for tumor immunotherapy, DEXs, such as AFP-enriched DEXs, induce a strong immune response with antigen specificity. A previous study showed that intravenous administration of exosomes from DEX_AFP_ into MHC-matched C57BL6 mice effectively suppress tumor growth by triggering a strong antigen-specific immune response and reshaping the tumor microenvironment. Therefore, the survival rates of mice with HCC were prolonged (25). Based on these results, DEX_AFP_ may be effective in patients with cancer, and we should explore the effect of DEX_AFP_ on patients with HCC. In addition, some phase I and II clinical trials based on DEXs for cancer have been completed and many of those trials documented the safety of DEXs, suggesting their potential in the development of cancer therapy (122) (**Figure 3**).

#### Perspectives for Clinical Application

Formed by the lipid bilayer membrane, exosomes protect their cargo from degradation by enzymes in the circulation (47). Additionally, exosomes have their own advantages, such as biocompatibility, low immunogenicity, the ability to penetrate the blood-brain barrier and an absence of inherent toxicity, enabling exosomes to become drug delivery vesicles (123). The results of some studies described above have shown the treatment effect of exosomes on animal models, including liver fibrosis, liver IRI, HCC and others (15, 23, 25). One study even found that exosomes from macrophages penetrate the blood-brain barrier and deliver a protein to the mouse brain, which indicated the feasibility of exosomes for delivering various materials (124). Furthermore, some studies have developed nanoparticles that encapsulate some substances and deliver them to target sites/cells (15). In addition, some clinical trials are exploring the possible clinical applications of exosomes (122, 125). All these results showed that exosomes are a feasible treatment or drug delivery tool in the clinic.

However, many limitations for the clinical application of exosomes still exist. Most of the experimental data described above were obtained from animal models and *in vitro* experiments. Some study did not obtain consistent results from animal experiments and *in vitro* experiments (15). As stated above, many exosome isolation methods have been developed, but each has merits and limitations. We may obtain unsatisfactory results even from the〝gold standard〞method –ultracentrifugation, since the type, quantity and quality are highly sensitive to multiple parameters (115). In addition, efficiently loading molecules into exosomes and providing clinical grade exosomes on a large scale may be obstacles for the clinical application of exosome therapy (125, 126). Furthermore, the optimal dose, the appropriate time window for exosome administration and route of administration that achieves maximal efficacy without adverse effects must be determined (127). There are a few clinical trials that assess the efficacy of exosome therapy, but few are conducted in liver diseases (121, 125, 128). More research is needed to study exosomes safety and effectiveness for clinical application in the future, especially in liver diseases.

## Conclusion

Exosomes are nanosized vesicles that mediate intercellular communication. Many immune cells produce exosomes that play a role in physiological and pathological conditions. Exosomes have been widely studied in various fields, and research investigating the role of exosomes derived from immune cells in liver diseases is gradually increasing. As described above, many clinical trials using DEXs as a therapy for cancer are in progress, such as those on non-small-cell lung cancer, advanced colorectal cancer and metastatic melanoma. Regarding liver diseases, many experiments have examined the effect of exosomes derived from immune cells on liver diseases. However, the mechanisms by which exosomes exert their effects on different disease states are not clear. Associated clinical trials on exosomes derived from immune cells are currently lacking. Moreover, the clinical application of exosomes is currently limited. From this perspective, the roles of exosomes derived from immune cells requires further investigation, especially those that have not yet been explored, and the identification of new biomarkers, such as exosomes and their cargoes, is warranted for the improved clinical treatment of liver diseases.

## Author Contributions

LZ and MS drafted the original manuscript and prepared the table and figure. LY conceived the idea. XF and YL provided critical feedback. All authors read and approved the submitted version.

## Funding

This work was supported by the National Natural Science Foundation of China (No. 82070582 to LY) and 1.3.5 Project for Disciplines of Excellence, West China Hospital, Sichuan University (No. 2019HXFH025 to LY).

## Conflict of Interest

The authors declare that the research was conducted in the absence of any commercial or financial relationships that could be construed as a potential conflict of interest.

## Publisher’s Note

All claims expressed in this article are solely those of the authors and do not necessarily represent those of their affiliated organizations, or those of the publisher, the editors and the reviewers. Any product that may be evaluated in this article, or claim that may be made by its manufacturer, is not guaranteed or endorsed by the publisher.

## Glossary

**Table:**

VH	viral hepatitis
HCC	hepatocellular carcinoma
DILI	drug-induced liver injury
TLR	Toll-like receptor
ALD	alcoholic liver disease
AIH	autoimmune hepatitis
PBC	primary biliary cirrhosis
IRI	ischemia/reperfusion injury
NASH	nonalcoholic steatohepatitis
EV	extracellular vesicles
MVEs	multivesicular endosomes
imDCs	immature dendritic cells
MHC	major histocompatibility complex
HAV	hepatitis A virus
ALI	acute liver injury
LPS	lipopolysaccharide
ALT	alanine aminotransferase
AST	aspartate transaminase
Con A	concanavalin A
HBV	hepatitis B virus
IFN-α	interferon-α
TIM-1	T cell immunoglobulin and mucin receptor 1
HCV	hepatitis C virus
AH	alcoholic hepatitis
TREM2	triggering receptor expressed on myeloid cells-2
NAFLD	nonalcoholic fatty liver disease
Mfn2	mitofusin 2
NAS	NAFLD activity score
ATMs	adipose tissue macrophages
HSCs	hepatic stellate cells
KLF4	Krüppel-like factor 4
PA	palmitic acid
TAZ	transcriptional activator with PDZ-binding motif
ASK1	apoptosis signal-regulating kinase 1
PPAR-γ	peroxisome proliferator-activated receptor-γ
AR	androgen receptor
TAMs	tumor-associated macrophages
LATS1	large tumor suppressor kinase 1
M2-TAMs	M2-polarized tumor-associated macrophages
BMDC	bone marrow-derived dendritic cell
HSP70	heat shock protein 70
DEXs	exosomes derived from dendritic cells
imDEXs	exosomes from immature dendritic cells
AFP	alpha fetoprotein
DEXAFP	exosomes released by α-fetoprotein (AFP)-expressing dendritic cells
NK-Exos	exosomes released by natural killer cells
ATG7	autophagy-related protein 7
APOE	apolipoprotein E
LDLR	low-density lipoprotein receptor
IHH	Indian hedgehog
OLT	orthotopic liver transplantation
RA	rheumatoid arthritis
MCs	mast cells
HCV-E2	HCV-E2 envelope glycoprotein
ALI	acute liver injury
DILI	drug-induced liver injury
Poly I:C	polyinosinic-polycytidylic acid
PA	palmitic acid
PMA	phorbol-12-myristate-13-acetate
CTLs	cytotoxic T lymphocytes
MVB	multivesicular bodies
RER	rough endoplasmic reticulum

## References

[1] Asrani SK Devarbhavi H Eaton J Kamath PS. Burden of Liver Diseases in the World. J Hepatol (2019) 70(1):151–71. doi: 10.1016/j.jhep.2018.09.014 30266282

[2] Heymann F Tacke F. Immunology in the Liver–From Homeostasis to Disease. Nat Rev Gastroenterol Hepatol (2016) 13(2):88–110. doi: 10.1038/nrgastro.2015.200 26758786

[3] Li W Li C Zhou T Liu X Liu X Li X. Role of Exosomal Proteins in Cancer Diagnosis. Mol Cancer (2017) 16(1):145. doi: 10.1186/s12943-017-0706-8 28851367 PMC5576100

[4] Nojima H Freeman CM Schuster RM Japtok L Kleuser B Edwards MJ. Hepatocyte Exosomes Mediate Liver Repair and Regeneration *via* Sphingosine-1-Phosphate. J Hepatol (2016) 64(1):60–8. doi: 10.1016/j.jhep.2015.07.030 PMC4843792 26254847

[5] Li J Liu K Liu Y Xu Y Zhang F Yang H. Exosomes Mediate the Cell-To-Cell Transmission of IFN-α-Induced Antiviral Activity. Nat Immunol (2013) 14(8):793–803. doi: 10.1038/ni.2647 23832071

[6] Verma VK Li H Wang R Hirsova P Mushref M Liu Y. Alcohol Stimulates Macrophage Activation Through Caspase-Dependent Hepatocyte Derived Release of CD40L Containing Extracellular Vesicles. J Hepatol (2016) 64(3):651–60. doi: 10.1016/j.jhep.2015.11.020 PMC4761285 26632633

[7] Hirsova P Ibrahim SH Krishnan A Verma VK Bronk SF Werneburg NW. Lipid-Induced Signaling Causes Release of Inflammatory Extracellular Vesicles From Hepatocytes. Gastroenterology (2016) 150(4):956–67. doi: 10.1053/j.gastro.2015.12.037 PMC4808464 26764184

[8] Gao J Wei B de Assuncao TM Liu Z Hu X Ibrahim S. Hepatic Stellate Cell Autophagy Inhibits Extracellular Vesicle Release to Attenuate Liver Fibrosis. J Hepatol (2020) 73(5):1144–54. doi: 10.1016/j.jhep.2020.04.044 PMC7572579 32389810

[9] Li R Wang Y Zhang X Feng M Ma J Li J. Exosome-Mediated Secretion of LOXL4 Promotes Hepatocellular Carcinoma Cell Invasion and Metastasis. Mol Cancer (2019) 18(1):18. doi: 10.1186/s12943-019-0948-8 30704479 PMC6354392

[10] Saha B Momen-Heravi F Kodys K Szabo G. MicroRNA Cargo of Extracellular Vesicles From Alcohol-Exposed Monocytes Signals Naive Monocytes to Differentiate Into M2 Macrophages. J Biol Chem (2016) 291(1):149–59. doi: 10.1074/jbc.M115.694133 PMC4697152 26527689

[11] Wang G Jin S Ling X Li Y Hu Y Zhang Y. Proteomic Profiling of LPS-Induced Macrophage-Derived Exosomes Indicates Their Involvement in Acute Liver Injury. Proteomics (2019) 19(3):e1800274. doi: 10.1002/pmic.201800274 30474914

[12] Kawata R Oda S Koya Y Kajiyama H Yokoi T. Macrophage-Derived Extracellular Vesicles Regulate Concanavalin A-Induced Hepatitis by Suppressing Macrophage Cytokine Production. Toxicology (2020) 443:152544. doi: 10.1016/j.tox.2020.152544 32739513

[13] Chen L Yao X Yao H Ji Q Ding G Liu X. Exosomal miR-103-3p From LPS-Activated THP-1 Macrophage Contributes to the Activation of Hepatic Stellate Cells. FASEB J (2020) 34(4):5178–92. doi: 10.1096/fj.201902307RRR 32061112

[14] Hou X Yin S Ren R Liu S Yong L Liu Y. Myeloid-Cell-Specific IL-6 Signaling Promotes MicroRNA-223-Enriched Exosome Production to Attenuate NAFLD-Associated Fibrosis. Hepatology (2021) 74(1):116–32. doi: 10.1002/hep.31658 PMC8141545 33236445

[15] Hu M Wang Y Liu Z Yu Z Guan K Liu M. Hepatic Macrophages Act as a Central Hub for Relaxin-Mediated Alleviation of Liver Fibrosis. Nat Nanotechnol (2021) 16(4):466–77. doi: 10.1038/s41565-020-00836-6 33495618

[16] Yao Z Qiao Y Li X Chen J Ding J Bai L. Exosomes Exploit the Virus Entry Machinery and Pathway To Transmit Alpha Interferon-Induced Antiviral Activity. J Virol (2018) 92(24):e01578–18. doi: 10.1128/JVI.01578-18 30282711 PMC6258946

[17] Zhou Y Wang X Sun L Zhou L Ma TC Song L. Toll-Like Receptor 3-Activated Macrophages Confer Anti-HCV Activity to Hepatocytes Through Exosomes. FASEB J (2016) 30(12):4132–40. doi: 10.1096/fj.201600696R PMC5102108 27605546

[18] Hou J Zhang J Cui P Zhou Y Liu C Wu X. TREM2 Sustains Macrophage-Hepatocyte Metabolic Coordination in Nonalcoholic Fatty Liver Disease and Sepsis. J Clin Invest (2021) 131(4):e135197. doi: 10.1172/JCI135197 PMC7880419 33586673

[19] Liu G Ouyang X Sun Y Xiao Y You B Gao Y. The miR-92a-2-5p in Exosomes From Macrophages Increases Liver Cancer Cells Invasion *via* Altering the AR/PHLPP/p-AKT/β-Catenin Signaling. Cell Death Differ (2020) 27(12):3258–72. doi: 10.1038/s41418-020-0575-3 PMC7853149 32587378

[20] Wang Y Wang B Xiao S Li Y Chen Q. miR-125a/B Inhibits Tumor-Associated Macrophages Mediated in Cancer Stem Cells of Hepatocellular Carcinoma by Targeting CD90. J Cell Biochem (2019) 120(3):3046–55. doi: 10.1002/jcb.27436 30536969

[21] Li J Xue J Ling M Sun J Xiao T Dai X. MicroRNA-15b in Extracellular Vesicles From Arsenite-Treated Macrophages Promotes the Progression of Hepatocellular Carcinomas by Blocking the LATS1-Mediated Hippo Pathway. Cancer Lett (2021) 497:137–53. doi: 10.1016/j.canlet.2020.10.023 33080309

[22] Ning J Ye Y Bu D Zhao G Song T Liu P. Imbalance of TGF-β1/BMP-7 Pathways Induced by M2-Polarized Macrophages Promotes Hepatocellular Carcinoma Aggressiveness. Mol Ther J Am Soc Gene Ther (2021) 29(6):2067–87. doi: 10.1016/j.ymthe.2021.02.016 PMC8178441 33601054

[23] Zheng L Li Z Ling W Zhu D Feng Z Kong L. Exosomes Derived From Dendritic Cells Attenuate Liver Injury by Modulating the Balance of Treg and Th17 Cells After Ischemia Reperfusion. Cell Physiol Biochem Int J Exp Cell Physiol Biochem Pharmacol (2018) 46(2):740–56. doi: 10.1159/000488733 29621784

[24] Ma B Yang JY Song WJ Ding R Zhang ZC Ji HC. Combining Exosomes Derived From Immature DCs With Donor Antigen-Specific Treg Cells Induces Tolerance in a Rat Liver Allograft Model. Sci Rep (2016) 6:32971. doi: 10.1038/srep32971 27640806 PMC5027549

[25] Lu Z Zuo B Jing R Gao X Rao Q Liu Z. Dendritic Cell-Derived Exosomes Elicit Tumor Regression in Autochthonous Hepatocellular Carcinoma Mouse Models. J Hepatol (2017) 67(4):739–48. doi: 10.1016/j.jhep.2017.05.019 28549917

[26] Wang L Wang Y Quan J. Exosomes Derived From Natural Killer Cells Inhibit Hepatic Stellate Cell Activation and Liver Fibrosis. Hum Cell (2020) 33(3):582–9. doi: 10.1007/s13577-020-00371-5 32449114

[27] Wang L Wang Y Quan J. Exosomal miR-223 Derived From Natural Killer Cells Inhibits Hepatic Stellate Cell Activation by Suppressing Autophagy. Mol Med (Cambridge Mass) (2020) 26(1):81. doi: 10.1186/s10020-020-00207-w 32873229 PMC7465359

[28] He Y Rodrigues RM Wang X Seo W Ma J Hwang S. Neutrophil-To-Hepatocyte Communication *via* LDLR-Dependent miR-223-Enriched Extracellular Vesicle Transfer Ameliorates Nonalcoholic Steatohepatitis. J Clin Invest (2021) 131(3):e141513. doi: 10.1172/JCI141513 PMC7843220 33301423

[29] Chen L Huang H Zhang W Ding F Fan Z Zeng Z. Exosomes Derived From T Regulatory Cells Suppress CD8+ Cytotoxic T Lymphocyte Proliferation and Prolong Liver Allograft Survival. Med Sci Monit Int Med J Exp Clin Res (2019) 25:4877–84. doi: 10.12659/MSM.917058 PMC6618337 31258152

[30] Liao TL Hsieh SL Chen YM Chen HH Liu HJ Lee HC. Rituximab May Cause Increased Hepatitis C Virus Viremia in Rheumatoid Arthritis Patients Through Declining Exosomal MicroRNA-155. Arthritis Rheumatol (Hoboken NJ) (2018) 70(8):1209–19. doi: 10.1002/art.40495 29575671

[31] Xiong L Zhen S Yu Q Gong Z. HCV-E2 Inhibits Hepatocellular Carcinoma Metastasis by Stimulating Mast Cells to Secrete Exosomal Shuttle microRNAs. Oncol Lett (2017) 14(2):2141–6. doi: 10.3892/ol.2017.6433 PMC5530191 28781655

[32] van Niel G D'Angelo G Raposo G. Shedding Light on the Cell Biology of Extracellular Vesicles. Nat Rev Mol Cell Biol (2018) 19(4):213–28. doi: 10.1038/nrm.2017.125 29339798

[33] Yáñez-Mó M Siljander PR Andreu Z Zavec AB Borràs FE Buzas EI. Biological Properties of Extracellular Vesicles and Their Physiological Functions. J Extracell Vesicles (2015) 4:27066. doi: 10.3402/jev.v4.27066 25979354 PMC4433489

[34] Théry C Ostrowski M Segura E. Membrane Vesicles as Conveyors of Immune Responses. Nat Rev Immunol (2009) 9(8):581–93. doi: 10.1038/nri2567 19498381

[35] EL Andaloussi S Mäger I Breakefield XO Wood MJ. Extracellular Vesicles: Biology and Emerging Therapeutic Opportunities. Nat Rev Drug Discov (2013) 12(5):347–57. doi: 10.1038/nrd3978 23584393

[36] Thompson AG Gray E Heman-Ackah SM Mäger I Talbot K Andaloussi SE. Extracellular Vesicles in Neurodegenerative Disease - Pathogenesis to Biomarkers. Nat Rev Neurol (2016) 12(6):346–57. doi: 10.1038/nrneurol.2016.68 27174238

[37] Mathieu M Martin-Jaular L Lavieu G Théry C. Specificities of Secretion and Uptake of Exosomes and Other Extracellular Vesicles for Cell-To-Cell Communication. Nat Cell Biol (2019) 21(1):9–17. doi: 10.1038/s41556-018-0250-9 30602770

[38] Théry C Witwer KW Aikawa E Alcaraz MJ Anderson JD Andriantsitohaina R. Minimal Information for Studies of Extracellular Vesicles 2018 (MISEV2018): A Position Statement of the International Society for Extracellular Vesicles and Update of the MISEV2014 Guidelines. J Extracell Vesicles (2018) 7(1):1535750. doi: 10.1080/20013078.2018.1535750 30637094 PMC6322352

[39] Pegtel DM Gould SJ. Exosomes. Annu Rev Biochem (2019) 88:487–514. doi: 10.1146/annurev-biochem-013118-111902 31220978

[40] Tkach M Théry C. Communication by Extracellular Vesicles: Where We Are and Where We Need to Go. Cell (2016) 164(6):1226–32. doi: 10.1016/j.cell.2016.01.043 26967288

[41] Kourembanas S. Exosomes: Vehicles of Intercellular Signaling, Biomarkers, and Vectors of Cell Therapy. Annu Rev Physiol (2015) 77:13–27. doi: 10.1146/annurev-physiol-021014-071641 25293529

[42] Segura E Amigorena S Théry C. Mature Dendritic Cells Secrete Exosomes With Strong Ability to Induce Antigen-Specific Effector Immune Responses. Blood Cells Mol Dis (2005) 35(2):89–93. doi: 10.1016/j.bcmd.2005.05.003 15990342

[43] Mittelbrunn M Gutiérrez-Vázquez C Villarroya-Beltri C González S Sánchez-Cabo F González M. Unidirectional Transfer of microRNA-Loaded Exosomes From T Cells to Antigen-Presenting Cells. Nat Commun (2011) 2:282. doi: 10.1038/ncomms1285 21505438 PMC3104548

[44] Zipkin M. Big Pharma Buys Into Exosomes for Drug Delivery. Nat Biotechnol (2020) 38(11):1226–8. doi: 10.1038/s41587-020-0725-7 33144725

[45] Costafreda MI Abbasi A Lu H Kaplan G. Exosome Mimicry by a HAVCR1-NPC1 Pathway of Endosomal Fusion Mediates Hepatitis A Virus Infection. Nat Microbiol (2020) 5(9):1096–106. doi: 10.1038/s41564-020-0740-y PMC7483988 32541946

[46] Hoshino A Kim HS Bojmar L Gyan KE Cioffi M Hernandez J. Extracellular Vesicle and Particle Biomarkers Define Multiple Human Cancers. Cell (2020) 182(4):1044–61.e18. doi: 10.1016/j.cell.2020.07.009 32795414 PMC7522766

[47] LeBleu VS Kalluri R. Exosomes as a Multicomponent Biomarker Platform in Cancer. Trends Cancer (2020) 6(9):767–74. doi: 10.1016/j.trecan.2020.03.007 32307267

[48] Lemprière S. Exosomal α-Synuclein as a Biomarker for Parkinson Disease. Nat Rev Neurol (2020) 16(5):242–3. doi: 10.1038/s41582-020-0349-z 32203397

[49] Mulcahy LA Pink RC Carter DR. Routes and Mechanisms of Extracellular Vesicle Uptake. J Extracell Vesicles (2014) 3:10.3402/jev.v3.24641. doi: 10.3402/jev.v3.24641 PMC4122821 25143819

[50] Barrès C Blanc L Bette-Bobillo P André S Mamoun R Gabius HJ. Galectin-5 is Bound Onto the Surface of Rat Reticulocyte Exosomes and Modulates Vesicle Uptake by Macrophages. Blood (2010) 115(3):696–705. doi: 10.1182/blood-2009-07-231449 19903899

[51] Geissmann F Manz MG Jung S Sieweke MH Merad M Ley K. Development of Monocytes, Macrophages, and Dendritic Cells. Science (New York NY) (2010) 327(5966):656–61. doi: 10.1126/science.1178331 PMC2887389 20133564

[52] Guilliams M Mildner A Yona S. Developmental and Functional Heterogeneity of Monocytes. Immunity (2018) 49(4):595–613. doi: 10.1016/j.immuni.2018.10.005 30332628

[53] Sica A Larghi P Mancino A Rubino L Porta C Totaro MG. Macrophage Polarization in Tumour Progression. Semin Cancer Biol (2008) 18(5):349–55. doi: 10.1016/j.semcancer.2008.03.004 18467122

[54] Wynn TA Chawla A Pollard JW. Macrophage Biology in Development, Homeostasis and Disease. Nature (2013) 496(7446):445–55. doi: 10.1038/nature12034 PMC3725458 23619691

[55] Cai C Koch B Morikawa K Suda G Sakamoto N Rueschenbaum S. Macrophage-Derived Extracellular Vesicles Induce Long-Lasting Immunity Against Hepatitis C Virus Which Is Blunted by Polyunsaturated Fatty Acids. Front Immunol (2018) 9:723. doi: 10.3389/fimmu.2018.00723 29706960 PMC5906748

[56] Galindo-Hernández O Córdova-Guerrero I Díaz-Rubio LJ Pulido-Capiz Á Díaz-Villanueva JF Castañeda-Sánchez CY. Protein Translation Associated to PERK Arm is a New Target for Regulation of Metainflammation: A Connection With Hepatocyte Cholesterol. J Cell Biochem (2019) 120(3):4158–71. doi: 10.1002/jcb.27701 30320914

[57] Li J Liu H Mauer AS Lucien F Raiter A Bandla H. Characterization of Cellular Sources and Circulating Levels of Extracellular Vesicles in a Dietary Murine Model of Nonalcoholic Steatohepatitis. Hepatol Commun (2019) 3(9):1235–49. doi: 10.1002/hep4.1404 PMC6719742 31497744

[58] Ying W Riopel M Bandyopadhyay G Dong Y Birmingham A Seo JB. Adipose Tissue Macrophage-Derived Exosomal miRNAs Can Modulate In Vivo and In Vitro Insulin Sensitivity. Cell (2017) 171(2):372–84.e12. doi: 10.1016/j.cell.2017.08.035 28942920

[59] Pu J Xu Z Nian J Fang Q Yang M Huang Y. M2 Macrophage-Derived Extracellular Vesicles Facilitate Cd8+T Cell Exhaustion in Hepatocellular Carcinoma *via* the miR-21-5p/YOD1/YAP/β-Catenin Pathway. Cell Death Discov (2021) 7(1):182. doi: 10.1038/s41420-021-00556-3 34282135 PMC8289864

[60] Kowal J Tkach M. Dendritic Cell Extracellular Vesicles. Int Rev Cell Mol Biol (2019) 349:213–49. doi: 10.1016/bs.ircmb.2019.08.005 31759432

[61] Gao W Liu H Yuan J Wu C Huang D Ma Y. Exosomes Derived From Mature Dendritic Cells Increase Endothelial Inflammation and Atherosclerosis *via* Membrane TNF-α Mediated NF-κb Pathway. J Cell Mol Med (2016) 20(12):2318–27. doi: 10.1111/jcmm.12923 PMC5134386 27515767

[62] Pang XL Wang ZG Liu L Feng YH Wang JX Xie HC. Immature Dendritic Cells Derived Exosomes Promotes Immune Tolerance by Regulating T Cell Differentiation in Renal Transplantation. Aging (2019) 11(20):8911–24. doi: 10.18632/aging.102346 PMC6834404 31655796

[63] Liu Q Rojas-Canales DM Divito SJ Shufesky WJ Stolz DB Erdos G. Donor Dendritic Cell-Derived Exosomes Promote Allograft-Targeting Immune Response. J Clin Invest (2016) 126(8):2805–20. doi: 10.1172/JCI84577 PMC4966303 27348586

[64] Collin M Ginhoux F. Human Dendritic Cells. Semin Cell Dev Biol (2019) 86:1–2. doi: 10.1016/j.semcdb.2018.04.015 29727728

[65] Puhr S Lee J Zvezdova E Zhou YJ Liu K. Dendritic Cell Development-History, Advances, and Open Questions. Semin Immunol (2015) 27(6):388–96. doi: 10.1016/j.smim.2016.03.012 PMC5032838 27040276

[66] Reis e Sousa C. Dendritic Cells in a Mature Age. Nat Rev Immunol (2006) 6(6):476–83. doi: 10.1038/nri1845 16691244

[67] Schraml BU Reis e Sousa C. Defining Dendritic Cells. Curr Opin Immunol (2015) 32:13–20. doi: 10.1016/j.coi.2014.11.001 25553392

[68] Wang Y Xiang Y Xin VW Wang XW Peng XC Liu XQ. Dendritic Cell Biology and its Role in Tumor Immunotherapy. J Hematol Oncol (2020) 13(1):107. doi: 10.1186/s13045-020-00939-6 32746880 PMC7397618

[69] Tkach M Kowal J Zucchetti AE Enserink L Jouve M Lankar D. Qualitative Differences in T-Cell Activation by Dendritic Cell-Derived Extracellular Vesicle Subtypes. EMBO J (2017) 36(20):3012–28. doi: 10.15252/embj.201696003 PMC5641679 28923825

[70] Zhong X Zhou Y Cao Y Ding J Wang P Luo Y. Enhanced Antitumor Efficacy Through Microwave Ablation Combined With a Dendritic Cell-Derived Exosome Vaccine in Hepatocellular Carcinoma. Int J Hyperthermia Off J Eur Soc Hyperthermic Oncol North Am Hyperthermia Group (2020) 37(1):1210–8. doi: 10.1080/02656736.2020.1836406 33100037

[71] Gillespie JR Uversky VN. Structure and Function of Alpha-Fetoprotein: A Biophysical Overview. Biochim Biophys Acta (2000) 1480(1-2):41–56. doi: 10.1016/S0167-4838(00)00104-7 11004554

[72] Yang X Chen J Wang N Liu Z Li Y. Clinical Use of Dendritic Cell-Derived Exosomes for Hepatocellular Carcinoma Immunotherapy: How Far We are? J Hepatol (2018) 69(4):984–6. doi: 10.1016/j.jhep.2018.07.003 30093161

[73] Caligiuri MA. Human Natural Killer Cells. Blood (2008) 112(3):461–9. doi: 10.1182/blood-2007-09-077438 PMC2481557 18650461

[74] Vivier E Tomasello E Baratin M Walzer T Ugolini S. Functions of Natural Killer Cells. Nat Immunol (2008) 9(5):503–10. doi: 10.1038/ni1582 18425107

[75] Freud AG Mundy-Bosse BL Yu J Caligiuri MA. The Broad Spectrum of Human Natural Killer Cell Diversity. Immunity (2017) 47(5):820–33. doi: 10.1016/j.immuni.2017.10.008 PMC5728700 29166586

[76] Silvestre-Roig C Fridlender ZG Glogauer M Scapini P. Neutrophil Diversity in Health and Disease. Trends Immunol (2019) 40(7):565–83. doi: 10.1016/j.it.2019.04.012 PMC7185435 31160207

[77] Amulic B Cazalet C Hayes GL Metzler KD Zychlinsky A. Neutrophil Function: From Mechanisms to Disease. Annu Rev Immunol (2012) 30:459–89. doi: 10.1146/annurev-immunol-020711-074942 22224774

[78] Cerwenka A Lanier LL. Natural Killer Cell Memory in Infection, Inflammation and Cancer. Nat Rev Immunol (2016) 16(2):112–23. doi: 10.1038/nri.2015.9 26806484

[79] Shimasaki N Jain A Campana D. NK Cells for Cancer Immunotherapy. Nat Rev Drug Discov (2020) 19(3):200–18. doi: 10.1038/s41573-019-0052-1 31907401

[80] de Oliveira S Rosowski EE Huttenlocher A. Neutrophil Migration in Infection and Wound Repair: Going Forward in Reverse. Nat Rev Immunol (2016) 16(6):378–91. doi: 10.1038/nri.2016.49 PMC5367630 27231052

[81] Genschmer KR Russell DW Lal C Szul T Bratcher PE Noerager BD. Activated PMN Exosomes: Pathogenic Entities Causing Matrix Destruction and Disease in the Lung. Cell J (2019) 176(1-2):113–26.e15. doi: 10.1016/j.cell.2018.12.002 PMC6368091 30633902

[82] Di Pace AL Tumino N Besi F Alicata C Conti LA Munari E. Characterization of Human NK Cell-Derived Exosomes: Role of DNAM1 Receptor In Exosome-Mediated Cytotoxicity Against Tumor. Cancers (2020) 12(3):661. doi: 10.3390/cancers12030661 PMC7140072 32178479

[83] Ravichandran G Neumann K Berkhout LK Weidemann S Langeneckert AE Schwinge D. Interferon-γ-Dependent Immune Responses Contribute to the Pathogenesis of Sclerosing Cholangitis in Mice. J Hepatol (2019) 71(4):773–82. doi: 10.1016/j.jhep.2019.05.023 31173810

[84] Hintermann E Bayer M Pfeilschifter JM Luster AD Christen U. CXCL10 Promotes Liver Fibrosis by Prevention of NK Cell Mediated Hepatic Stellate Cell Inactivation. J Autoimmun (2010) 35(4):424–35. doi: 10.1016/j.jaut.2010.09.003 PMC3855675 20932719

[85] Jeong WI Park O Radaeva S Gao B. STAT1 Inhibits Liver Fibrosis in Mice by Inhibiting Stellate Cell Proliferation and Stimulating NK Cell Cytotoxicity. Hepatol (Baltimore Md) (2006) 44(6):1441–51. doi: 10.1002/hep.21419 17133483

[86] Li X Zhang M Liu J Huang Z Zhao Q Huang Y. Intrahepatic NK Cells Function Suppressed in Advanced Liver Fibrosis. Eur J Clin Invest (2016) 46(10):864–72. doi: 10.1111/eci.12669 27555302

[87] Fan Y Zhang W Wei H Sun R Tian Z Chen Y. Hepatic NK Cells Attenuate Fibrosis Progression of Non-Alcoholic Steatohepatitis in Dependent of CXCL10-Mediated Recruitment. Liver Int (2020) 40(3):598–608. doi: 10.1111/liv.14307 31758647

[88] Liu M Hu Y Yuan Y Tian Z Zhang C. γδt Cells Suppress Liver Fibrosis *via* Strong Cytolysis and Enhanced NK Cell-Mediated Cytotoxicity Against Hepatic Stellate Cells. Front Immunol (2019) 10:477. doi: 10.3389/fimmu.2019.00477 30930903 PMC6428727

[89] Wang X Seo W Park SH Fu Y Hwang S Rodrigues RM. MicroRNA-223 Restricts Liver Fibrosis by Inhibiting the TAZ-IHH-GLI2 and PDGF Signaling Pathways *via* the Crosstalk of Multiple Liver Cell Types. Int J Biol Sci (2021) 17(4):1153–67. doi: 10.7150/ijbs.58365 PMC8040312 33867837

[90] Kumar BV Connors TJ Farber DL. Human T Cell Development, Localization, and Function Throughout Life. Immunity (2018) 48(2):202–13. doi: 10.1016/j.immuni.2018.01.007 PMC5826622 29466753

[91] LeBien TW Tedder TF. B Lymphocytes: How They Develop and Function. Blood (2008) 112(5):1570–80. doi: 10.1182/blood-2008-02-078071 PMC2518873 18725575

[92] Cai Z Yang F Yu L Yu Z Jiang L Wang Q. Activated T Cell Exosomes Promote Tumor Invasion *via* Fas Signaling Pathway. J Immunol (Baltimore Md 1950) (2012) 188(12):5954–61. doi: 10.4049/jimmunol.1103466 22573809

[93] Fu W Lei C Liu S Cui Y Wang C Qian K. CAR Exosomes Derived From Effector CAR-T Cells Have Potent Antitumour Effects and Low Toxicity. Nat Commun (2019) 10(1):4355. doi: 10.1038/s41467-019-12321-3 31554797 PMC6761190

[94] Kurachi M. CD8(+) T Cell Exhaustion. Semin Immunopathol (2019) 41(3):327–37. doi: 10.1007/s00281-019-00744-5 30989321

[95] Wang X Shen H He Q Tian W Xia A Lu XJ. Exosomes Derived From Exhausted CD8+ T Cells Impaired the Anticancer Function of Normal CD8+ T Cells. J Med Genet (2019) 56(1):29–31. doi: 10.1136/jmedgenet-2018-105439 29997128

[96] Momen-Heravi F Bala S Bukong T Szabo G. Exosome-Mediated Delivery of Functionally Active miRNA-155 Inhibitor to Macrophages. Nanomed Nanotechnol Biol Med (2014) 10(7):1517–27. doi: 10.1016/j.nano.2014.03.014 PMC4180003 24685946

[97] Piliponsky AM Romani L. The Contribution of Mast Cells to Bacterial and Fungal Infection Immunity. Immunol Rev (2018) 282(1):188–97. doi: 10.1111/imr.12623 PMC5812373 29431211

[98] Sáez T de Vos P Kuipers J Sobrevia L Faas MM. Exosomes Derived From Monocytes and From Endothelial Cells Mediate Monocyte and Endothelial Cell Activation Under High D-Glucose Conditions. Immunobiology (2019) 224(2):325–33. doi: 10.1016/j.imbio.2019.02.004 30827721

[99] Xie G Yang H Peng X Lin L Wang J Lin K. Mast Cell Exosomes can Suppress Allergic Reactions by Binding to IgE. J Allergy Clin Immunol (2018) 141(2):788–91. doi: 10.1016/j.jaci.2017.07.040 28916187

[100] Motawi TK Mohamed MR Shahin NN Ali MAM Azzam MA. Time-Course Expression Profile and Diagnostic Potential of a miRNA Panel in Exosomes and Total Serum in Acute Liver Injury. Int J Biochem Cell Biol (2018) 100:11–21. doi: 10.1016/j.biocel.2018.05.002 29738828

[101] Cho YE Kim SH Lee BH Baek MC. Circulating Plasma and Exosomal microRNAs as Indicators of Drug-Induced Organ Injury in Rodent Models. Biomol Ther (2017) 25(4):367–73. doi: 10.4062/biomolther.2016.174 PMC5499614 28208010

[102] Babuta M Furi I Bala S Bukong TN Lowe P Catalano D. Dysregulated Autophagy and Lysosome Function Are Linked to Exosome Production by Micro-RNA 155 in Alcoholic Liver Disease. Hepatol (Baltimore Md) (2019) 70(6):2123–41. doi: 10.1002/hep.30766 PMC7453183 31090940

[103] Brandon-Warner E Feilen NA Culberson CR Field CO deLemos AS Russo MW. Processing of Mir17-92 Cluster in Hepatic Stellate Cells Promotes Hepatic Fibrogenesis During Alcohol-Induced Injury. Alcoholism Clin Exp Res (2016) 40(7):1430–42. doi: 10.1111/acer.13116 PMC4930422 27291156

[104] Pu C Huang H Wang Z Zou W Lv Y Zhou Z. Extracellular Vesicle-Associated Mir-21 and Mir-144 Are Markedly Elevated in Serum of Patients With Hepatocellular Carcinoma. Front Physiol (2018) 9:930. doi: 10.3389/fphys.2018.00930 30065664 PMC6056643

[105] Kornek M Lynch M Mehta SH Lai M Exley M Afdhal NH. Circulating Microparticles as Disease-Specific Biomarkers of Severity of Inflammation in Patients With Hepatitis C or Nonalcoholic Steatohepatitis. Gastroenterology (2012) 143(2):448–58. doi: 10.1053/j.gastro.2012.04.031 PMC3404266 22537612

[106] Nielsen MC Andersen MN Grønbæk H Damgaard Sandahl T Møller HJ. Extracellular Vesicle-Associated Soluble CD163 and CD206 in Patients With Acute and Chronic Inflammatory Liver Disease. Scand J Gastroenterol (2020) 55(5):588–96. doi: 10.1080/00365521.2020.1759140 32393080

[107] Liu XL Pan Q Cao HX Xin FZ Zhao ZH Yang RX. Lipotoxic Hepatocyte-Derived Exosomal MicroRNA 192-5p Activates Macrophages Through Rictor/Akt/Forkhead Box Transcription Factor O1 Signaling in Nonalcoholic Fatty Liver Disease. Hepatol (Baltimore Md) (2020) 72(2):454–69. doi: 10.1002/hep.31050 PMC10465073 31782176

[108] Sehrawat TS Arab JP Liu M Amrollahi P Wan M Fan J. Circulating Extracellular Vesicles Carrying Sphingolipid Cargo for the Diagnosis and Dynamic Risk Profiling of Alcoholic Hepatitis. Hepatol (Baltimore Md) (2021) 73(2):571–85. doi: 10.1002/hep.31256 PMC7541595 32246544

[109] Niu LJ Zhang YM Huang T Sun XF Luo SX. Exosomal microRNA-155 as a Biomarker for Hepatic Fibrosis Diagnosis and Progression. Ann Trans Med (2021) 9(2):137. doi: 10.21037/atm-20-7787 PMC7867899 33569439

[110] Lee YR Kim G Tak WY Jang SY Kweon YO Park JG. Circulating Exosomal Noncoding RNAs as Prognostic Biomarkers in Human Hepatocellular Carcinoma. Int J Cancer (2019) 144(6):1444–52. doi: 10.1002/ijc.31931 30338850

[111] Fründt T Krause L Hussey E Steinbach B Köhler D von Felden J. Diagnostic and Prognostic Value of miR-16, miR-146a, miR-192 and miR-221 in Exosomes of Hepatocellular Carcinoma and Liver Cirrhosis Patients. Cancers (2021) 13(10):2484. doi: 10.3390/cancers13102484 34069692 PMC8161187

[112] Hwang S Yang YM. Exosomal microRNAs as Diagnostic and Therapeutic Biomarkers in non-Malignant Liver Diseases. Arch Pharmacal Res (2021) 44(6):574–87. doi: 10.1007/s12272-021-01338-2 PMC8223764 34165701

[113] Jiao Y Xu P Shi H Chen D Shi H. Advances on Liver Cell-Derived Exosomes in Liver Diseases. J Cell Mol Med (2021) 25(1):15–26. doi: 10.1111/jcmm.16123 33247543 PMC7810930

[114] Barile L Vassalli G. Exosomes: Therapy Delivery Tools and Biomarkers of Diseases. Pharmacol Ther (2017) 174:63–78. doi: 10.1016/j.pharmthera.2017.02.020 28202367

[115] Taylor DD Shah S. Methods of Isolating Extracellular Vesicles Impact Down-Stream Analyses of Their Cargoes. Methods (San Diego Calif) (2015) 87:3–10. doi: 10.1016/j.ymeth.2015.02.019 25766927

[116] Mercadal M Herrero C López-Rodrigo O Castells M de la Fuente A Vigués F. Impact of Extracellular Vesicle Isolation Methods on Downstream Mirna Analysis in Semen: A Comparative Study. Int J Mol Sci (2020) 21(17):5949. doi: 10.3390/ijms21175949 PMC7504614 32824915

[117] Santangelo L Bordoni V Montaldo C Cimini E Zingoni A Battistelli C. Hepatitis C Virus Direct-Acting Antivirals Therapy Impacts on Extracellular Vesicles microRNAs Content and on Their Immunomodulating Properties. Liver Int (2018) 38(10):1741–50. doi: 10.1111/liv.13700 29359389

[118] Viaud S Théry C Ploix S Tursz T Lapierre V Lantz O. Dendritic Cell-Derived Exosomes for Cancer Immunotherapy: What's Next? Cancer Res (2010) 70(4):1281–5. doi: 10.1158/0008-5472.CAN-09-3276 20145139

[119] Escudier B Dorval T Chaput N André F Caby MP Novault S. Vaccination of Metastatic Melanoma Patients With Autologous Dendritic Cell (DC) Derived-Exosomes: Results of Thefirst Phase I Clinical Trial. J Trans Med (2005) 3(1):10. doi: 10.1186/1479-5876-3-10 PMC554765 15740633

[120] Morse MA Garst J Osada T Khan S Hobeika A Clay TM. A Phase I Study of Dexosome Immunotherapy in Patients With Advanced non-Small Cell Lung Cancer. J Trans Med (2005) 3(1):9. doi: 10.1186/1479-5876-3-9 PMC551593 15723705

[121] Wang LT Liu KJ Sytwu HK Yen ML Yen BL. Advances in Mesenchymal Stem Cell Therapy for Immune and Inflammatory Diseases: Use of Cell-Free Products and Human Pluripotent Stem Cell-Derived Mesenchymal Stem Cells. Stem Cells Trans Med (2021) 10(9):1288–303. doi: 10.1002/sctm.21-0021 PMC8380447 34008922

[122] Pitt JM André F Amigorena S Soria JC Eggermont A Kroemer G. Dendritic Cell-Derived Exosomes for Cancer Therapy. J Clin Invest (2016) 126(4):1224–32. doi: 10.1172/JCI81137 PMC4811123 27035813

[123] Zhang S Hou Y Yang J Xie D Jiang L Hu H. Application of Mesenchymal Stem Cell Exosomes and Their Drug-Loading Systems in Acute Liver Failure. J Cell Mol Med (2020) 24(13):7082–93. doi: 10.1111/jcmm.15290 PMC7339207 32492261

[124] Yuan D Zhao Y Banks WA Bullock KM Haney M Batrakova E. Macrophage Exosomes as Natural Nanocarriers for Protein Delivery to Inflamed Brain. Biomaterials (2017) 142:1–12. doi: 10.1016/j.biomaterials.2017.07.011 28715655 PMC5603188

[125] Baghaei K Tokhanbigli S Asadzadeh H Nmaki S Reza Zali M Hashemi SM. Exosomes as a Novel Cell-Free Therapeutic Approach in Gastrointestinal Diseases. J Cell Physiol (2019) 234(7):9910–26. doi: 10.1002/jcp.27934 30536895

[126] Levine P McDaniel K Francis H Kennedy L Alpini G Meng F. Molecular Mechanisms of Stem Cell Therapy in Alcoholic Liver Disease. Digest Liver Dis Off J Ital Soc Gastroenterol Ital Assoc Study Liver (2014) 46(5):391–7. doi: 10.1016/j.dld.2013.11.015 24440312

[127] Cheng L Zhang K Wu S Cui M Xu T. Focus on Mesenchymal Stem Cell-Derived Exosomes: Opportunities and Challenges in Cell-Free Therapy. Stem Cells Int (2017) 2017:6305295. doi: 10.1155/2017/6305295 29410682 PMC5749272

[128] Yin K Wang S Zhao RC. Exosomes From Mesenchymal Stem/Stromal Cells: A New Therapeutic Paradigm. Biomark Res (2019) 7:8. doi: 10.1186/s40364-019-0159-x 30992990 PMC6450000

